# PRE-OPERATIVE GASTRIC GIST DOWNSIZING: THE IMPORTANCE OF NEOADJUVANT THERAPY

**DOI:** 10.1590/0102-672020180001e1427

**Published:** 2019-02-07

**Authors:** João Bernardo Sancio Rocha RODRIGUES, Renato Gomes CAMPANATI, Francisco NOLASCO, Athos Miranda BERNARDES, Soraya Rodrigues de Almeida SANCHES, Paulo Roberto SAVASSI-ROCHA

**Affiliations:** 1 Hospital das Clínicas, Universidade Federal de Minas Gerais, Alfa Institute of Gastroenterology, Belo Horizonte, MG, Brazil

**Keywords:** Gastrointestinal stromal tumor, Neoadjuvant therap, Imatinib Mesylat, Molecular targeted therap, Chemotherapy, adjuvan, Tumores do estroma gastrointestina, Terapia neoadjuvant, Mesilato de imatini, Terapia de alvo molecula, Quimioterapia adjuvant

## Abstract

**Introduction:**

Gastric gastrointestinal tumors (GIST) are a rare and usually asymptomatic neoplasm that can present as abdominal mass in more advanced scenarios. Since surgical resection is the main aspect of the treatment, locally advanced tumors require multivisceral resection and, therefore, higher postoperative morbidity and mortality.

**Objective:**

To perform a review the literature on the topic, with emphasis on the neoadjuvant therapy.

**Methods:**

Literature review on the Medline database using the following descriptors: gastrointestinal stromal tumors, neoadjuvant therapy, imatinib mesylate and molecular targeted therapy.

**Results:**

Surgical resection remains the cornerstone for the treatment of GISTs; however, tyrosine kinase inhibitors have improved survival as an adjuvant therapy. More recently, neoadjuvant therapy have been described in the treatment of locally advanced tumors in order to avoid multivisceral resection.

**Conclusion:**

Despite surgical resection remains as the most important aspect of the treatment of GISTs, adjuvant and neoadjuvant therapy with tyrosine kinase inhibitors have shown to both improve survival and resectability, respectively.

## RESUMO

**Introdução:**

O tumor estromal gastrintestinal (GIST) gástrico é neoplasia que cursa, por vezes, com apresentação assintomática, se manifestando como massa abdominal, relacionada a casos mais avançados. Esses, por sua vez, exigem tratamento com operações maiores e que cursam com mais alta morbimortalidade.

**Objetivo:**

Revisar e atualizar o tratamento do GIST gástrico, com enfoque na relevância do tratamento neoadjuvante.

**Método:**

Revisão da literatura utilizando a base de dados Medline/PubMed. Utilizaram-se os seguintes descritores: gastrointestinal stromal tumors, neoadjuvant therapy, imatinib mesylate e molecular targeted therapy. Dos artigos selecionados, 20 foram incluídos.

**Resultados:**

O tratamento cirúrgico é pilar fundamental para a cura do GIST. Entretanto, após a introdução do mesilato de imatinibe, classicamente utilizado como terapia adjuvante, houve mudança no manejo do GIST, permitindo aumento da sobrevida e diminuição da recorrência. A aplicação como terapia neoadjuvante é mais recente, e visa evitar procedimentos maiores sem, no entanto, prejudicar o resultado oncológico.

**Conclusão:**

A ressecção cirúrgica possui papel bem estabelecido no tratamento do GIST, inclusive com abordagem laparoscópica. O tratamento adjuvante com mesilato de imatinib, durante os primeiros três anos após a operação, mostra-se como opção segura para casos com elevado risco de recidiva. A terapia neoadjuvante é opção promissora para casos de tumor localmente avançado, permitindo ressecções menores e com menor morbimortalidade operatória.

## INTRODUCTION

The gastrointestinal stromal tumor (GIST) is the most frequent mesenchymal tissue tumor of the gastrointestinal tract, accounting for up to 1 to 1.5 cases every 100.000 people/year, with a mean age of 60 years at diagnosis^16^. It originates from the Cajal cells, which are located at the muscle layers of the bowel wall and are involved in peristalsis^10^. Despite being also described outside the digestive tract, the most frequent location are the stomach (60% of cases) and the small bowel (around 20-30%)^15^ ^,^ ^16^. Usually asymptomatic, most of GISTs are an incidental diagnosis during surgical or image exams, but it can lead to abdominal swelling and pain in more advanced scenarios^16^ ^,^ ^17^. At the pathology exam, the defining factor for diagnosis other than morphological traits is the expression of the receptor of the proto-oncogene KIT (CD117)^16^.

Despite surgical resection is still considered the cornerstone of treatment, target therapies with tyrosine kinase inhibitors have also contributed to a greater improvement, since its use as adjuvant therapy have shown to increase overall and disease free-survival^1^ ^,^ ^4^ ^,^ ^7^ ^,^ ^11^ ^,^ ^16^. However, the preoperative therapy with such drugs can aid in specific cases with predicted higher morbidity, in order to enable more conservative surgical approaches and better oncological and functional results^1^.

## METHODS

This study was approved by ethical board of the institution and by the patient depicted with specific consent.

Literature review was performed using Medline database with the following descriptors: gastrointestinal stromal tumors, neoadjuvant therapy, imatinib mesylate and molecular targeted therapy.

## RESULTS

The imatinib mesylate is the first line of treatment for inoperable, recurred of metastatic GIST^4^ ^,^ ^16^. Surgical resection remains the best treatment for tumors that can be completely removed with free surgical margins^3^ ^,^ ^4^ ^,^ ^11^ ^,^ ^16^. Since lymphatic spread is rare, lymphadenectomy is not routinely perfomed^16^. Minimally invasive procedures are indicated, mainly in small gastric lesions, regarding its known benefits of early operative recovery and lower morbidity, but larger masses are less likely to be resected from laparoscopic approach due to higher perforation risk^4^ ^,^ ^9^ ^,^ ^11^ ^,^ ^12^ ^,^ ^16^.

Surgical resection with microscopically free margins is related with a 5-year overall survival rate of around 60%^16^. Since 2008, imatinib mesylate has been indicated as adjuvant therapy after surgical resection with a significant reduction in local recurrence^20^. A trial from DeMatteo *et al*.^2^ randomized patients with GIST of up to 3 cm after R0 resection to receive 400 mg/day of imatinib vs. placebo, showing a significant improvement in 1-year disease free-survival (98% vs. 83%, p<0,0001). Interestingly, overall survival rated did not differed between groups, probably due to short follow-up period.

Therefore, the definition of high-risk groups that are amenable to adjuvant therapy is of foremost importance and classically it takes into account the mitotic rate, tumor size and primary site, as well as tumor perforation during surgery^4^. Regarding these factors, several classification systems have been proposed, including that of Miettinen and Lasota^13^ that stratifies patients as very low risk, low risk, intermediate risk and high risk of postoperative progression^3^ ^-^ ^5^ ^,^ ^13^ ^,^ ^14^. On the other hand, the 2016 Asian consensus^11^ adopts the classification by Joensuu^8^, from 2008. High risk tumors, regarding the Joensuu classification, are amenable to adjuvant therapy with 400 mg of imatinib mesylate (Table 1).

**TABLE 1:** Prognostic classification of recurrence risk for the selection of adjuvant therapy in patients with GIST*

Risk category	Tumor size in largest dimension	Mitotic count (per 50 HPFs§)	Primary site
Very low risk	<2 cm	≤5	Any
Low risk	>2 and ≤5 cm	≤5	Any
Intermediate risk	>2 and ≤5 cm	>5	Gastric
	<5 cm	>5 and ≤10	Any
	>5 and ≤10 cm	≤5	Gastric
High risk	Tumoral Rupture		
	>10 cm	Any	Any
	Any	>10	Any
	>5 cm	>5	Any
	>2 and ≤5 cm	>5	Non-gastric
	>5 and ≤10 cm	≤5	Non-gastric

* Adapted from Joensuu^8^; ^§^ number of mitosis per 50 high-power fields.

Therefore, it is defined that patients with gastric GIST that should receive adjuvant therapy with imatinib mesylate, 400 mg/day, are those in which there was tumor rupture during intraoperative time, tumors greater than 10 cm or with a mitotic count greater than 10 mitosis per 50 high-power fields (HPFs), as well as those larger than 5 cm associated with a mitotic count greater than five mitosis per 50 HPFs^11^.

Regarding the extent of the adjuvant therapy, another multicentric randomized trial compared 1 vs. 3 years of duration and demonstrated a greater 5-year disease free and overall survival for the longer duration group (47,9 vs. 65,6%, p<0,001 and 81,7 vs. 92,0%, p=0,02, respectively)^6^. Therefore, if adjuvant therapy is indicated, it should be performed for three years and, according to the main current consensus, its initiation should occur as soon as possible after the operation, once the patient has oral intake^4^ ^,^ ^11^.

More recently, neoadjuvant therapy has been considered in cases of locally advanced tumors, where is predicted positive resection margins and, therefore, a higher chance of bleeding and perforation. Other than that it can also be indicated to avoid multivisceral resections in order to minimize postoperative morbidity and to enable the surgical approach^1^ ^,^ ^4^ ^,^ ^11^ ^,^ ^19^ ^,^ ^20^. 

Gene sequencing methods are indicated before the therapy since it can predict response and the most common ones are located at the KIT gene in exons 11 (65%) and 9 (8%). Exons 11 and 13 mutations in this gene is associated with better response and prognosis and the exon 9 mutation, on the other hand, with lower response to imatinib and more aggressive tumors. The mutation on the gene PDGFRA (D8842V) and the other 10% of tumors that does not present with any other mutations show minimal or no response after tyrosine kinase inhibitors therapy, hence the importance of gene sequencing^16^.

**FIGURE 1:**
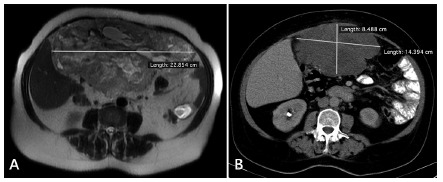
A) Magnetic resonance imaging of the abdomen before neoadjuvant therapy showing a tumor with 22,8 cm in its greater diameter; B) computed tomographic image after neoadjuvant therapy.

Neoadjuvant therapy can be maintained for 4-12 months and does not require preemptive suspension before surgical approach^4^ ^,^ ^11^. Usually, imaging exams are repeated after the first month of therapy, specially when gene sequencing was not performed, in order to detect non-response (Figure 1)^4^ ^,^ ^11^.

There is no specific criteria to measure tumor response on image exams and, particularly, to determine the behavior of the GIST. 

Another interesting finding is an unexpectedly good response of some patients with metastatic disease after imatinib therapy making it even amenable to surgical resection^18^.

To this very moment, there is no good quality evidence on the follow up of patients with GISTs after surgical resection and most of the data are based on expert opinion. Since extra-abdominal metastatic dissemination is quite uncommon in gastrointestinal stromal tumors, computed tomography (CT) of the abdomen and pelvis appears to be sufficient as a method of imaging during follow-up, and may be replaced by magnetic resonance imaging in younger patients, in order to decrease exposure to excessive radiation. For intermediate or low-risk patients, an annual CT scan during the first five years after resection is considered adequate. The typical recommendation for high-risk patients is to perform an image exam every six months in the first two years and subsequently every 6-12 months^4^ ^,^ ^11^ ^,^ ^16^. 

## CONCLUSION

Surgical resection of gastric GIST remains the cornerstone of the treatment of these tumors, with minimally invasive approaches being the usual choice whenever possible. Adjuvant therapy with tyrosine kinase inhibitors is indicated in high-risk patients in the first three years after operation and, more recently, neoadjuvant therapy presents as a reasonable option in locally advanced tumors, in order to reduce postoperative morbidity and to increase resectability.
